# Supplementary figures and images for: Exploring vaccine hesitancy in digital public discourse: From tribal polarization to socio-economic disparities
Source: PLoS One. 2024 Nov 5;19(11):e0308122. doi: 10.1371/journal.pone.0308122 (PMC11537378; doi:10.1371/journal.pone.0308122)

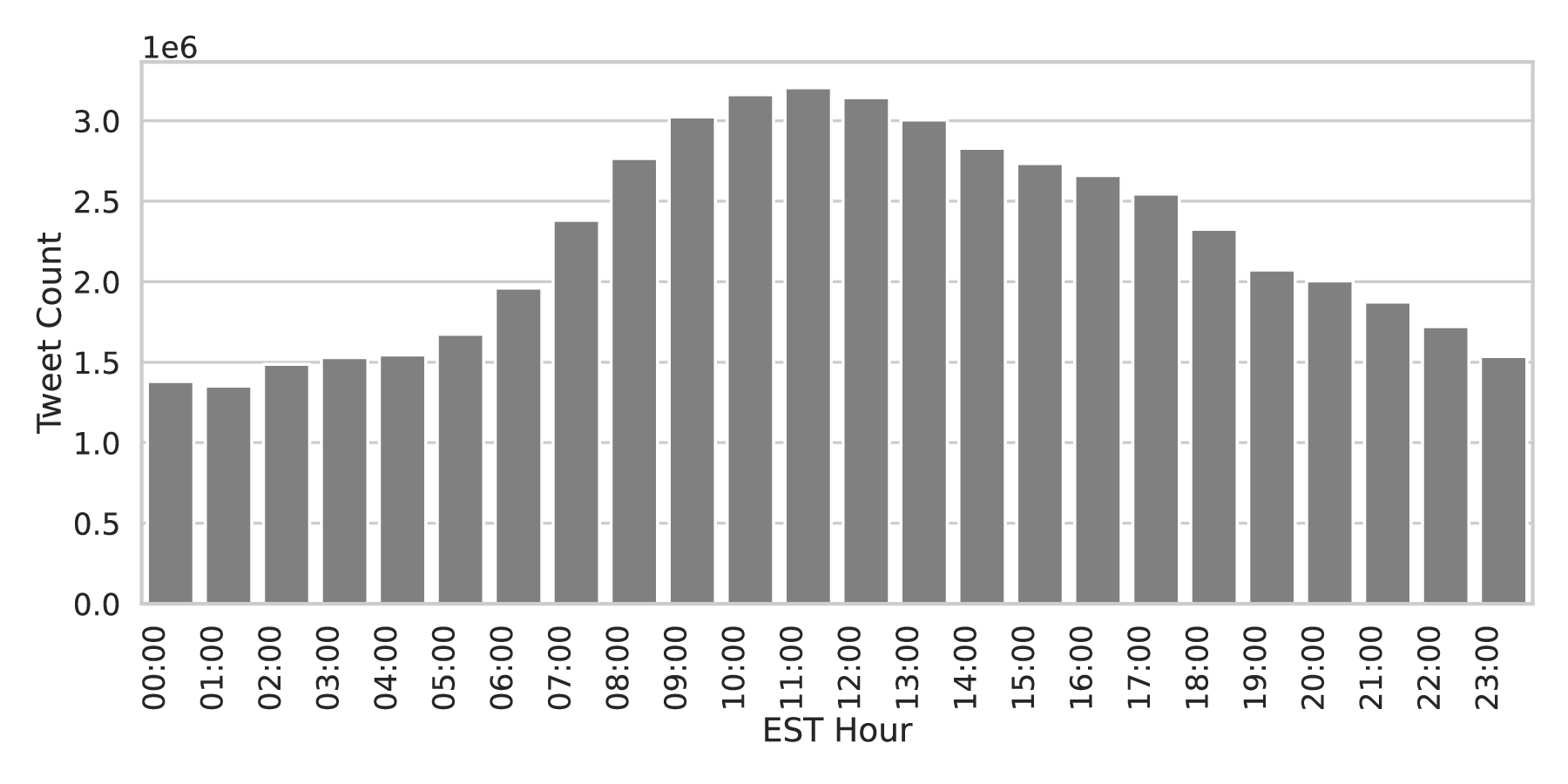

Supplement: S1 Fig — The results show that the number of tweets peaks during the daytime hours of EST. (TIFF) [file pone.0308122.s001.tiff]

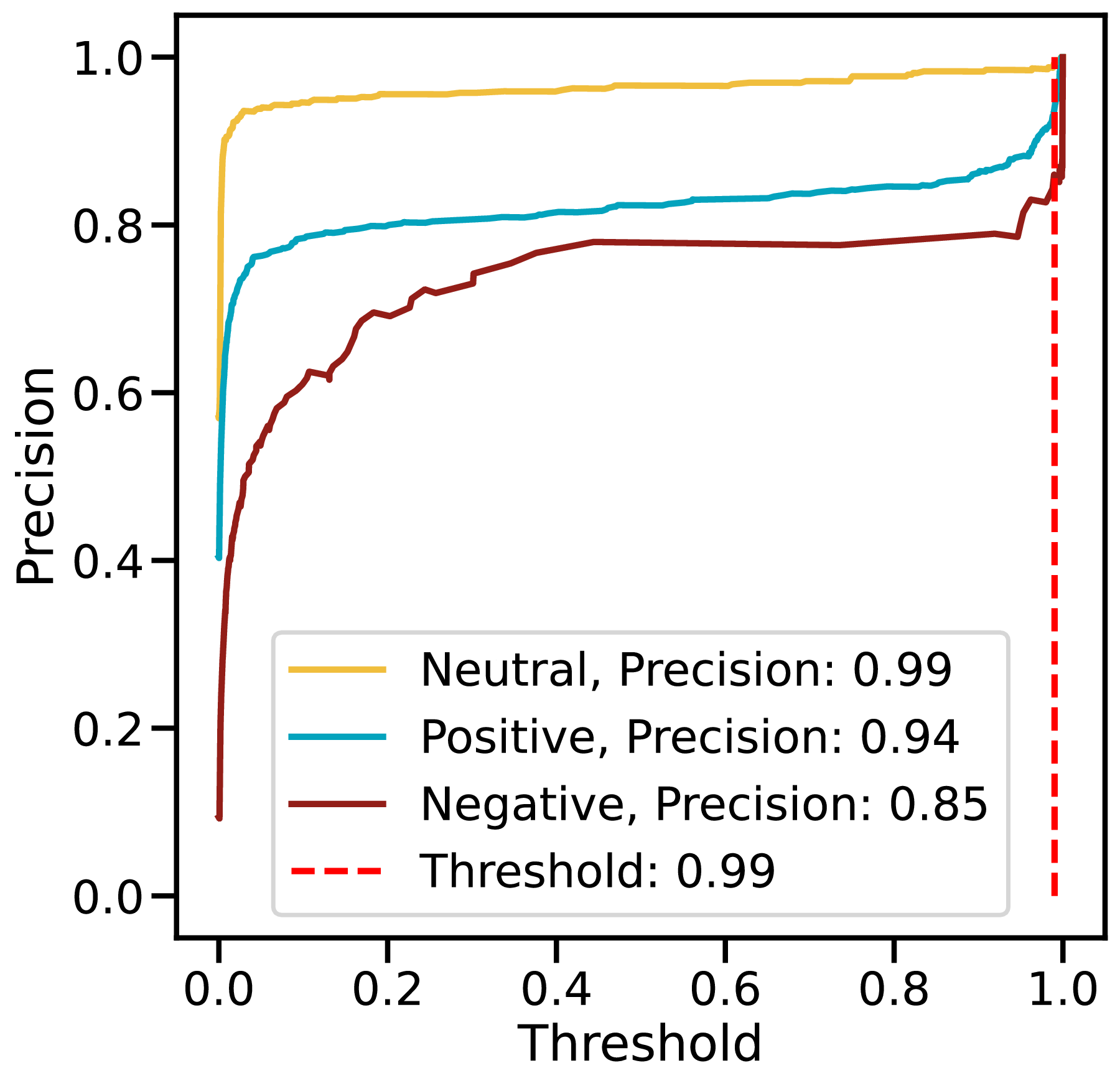

Supplement: S2 Fig — Precision is a measure of correctly classified samples out of all samples classified as a specific class, while the threshold is the minimum class probability required for a class assignment. The dashed red line represents a threshold of 0.99. (TIFF) [file pone.0308122.s002.tiff]

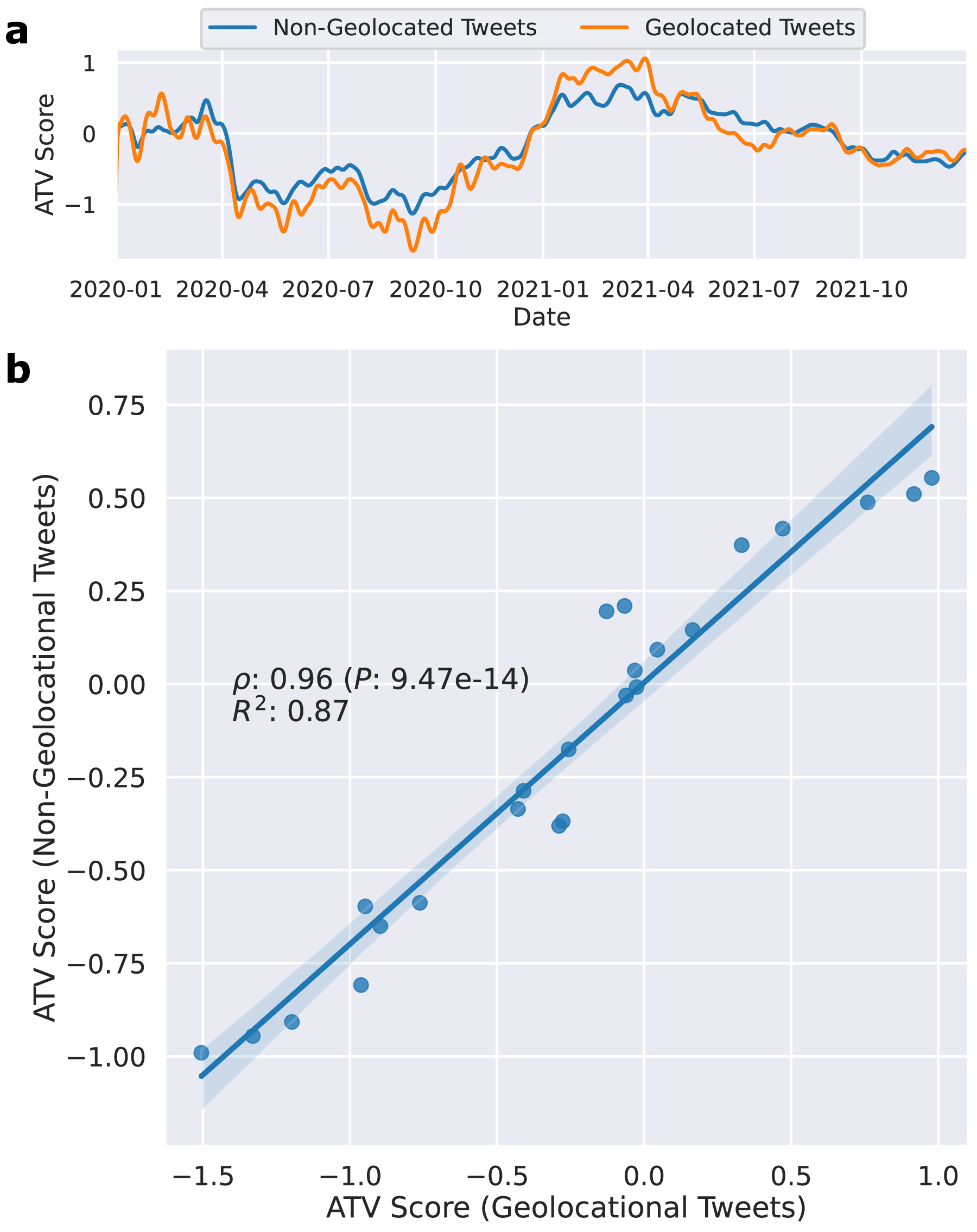

Supplement: S3 Fig — A: This figure displays the daily ATV score for geolocated and non-geolocated tweets between 2020 and 2022. Geolocated and non-geolocated tweets follow the same trend. B: Monthly ATV scores correlation between geolocated and non-geolocated tweets. There is a high correlation (R-squared of 87%) between ATV scores of geolocated and non-geolocated tweets. (TIFF) [file pone.0308122.s003.tiff]

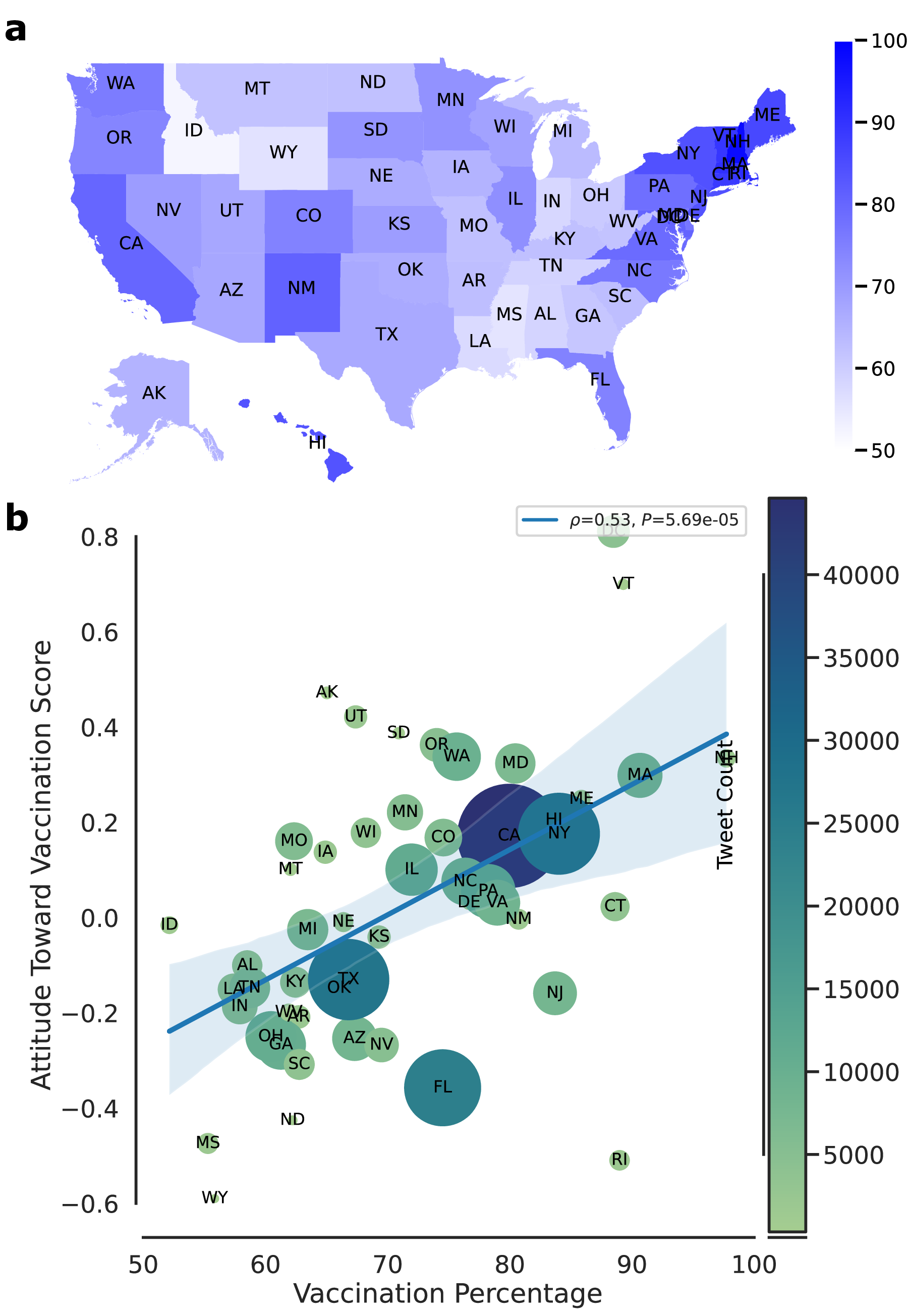

Supplement: S4 Fig — A: The map displays the vaccination percentage of US states as of December 31st, 2021. B: This scatter plot presents the correlation between the ATV score of each state and the percentage of eligible population vaccinated in the state. A moderate Spearman’s correlation coefficient indicates that attitude on social media is predictive of the actual vaccination rate. The size and color of the points represent the tweet count of each state. (TIFF) [file pone.0308122.s004.tiff]

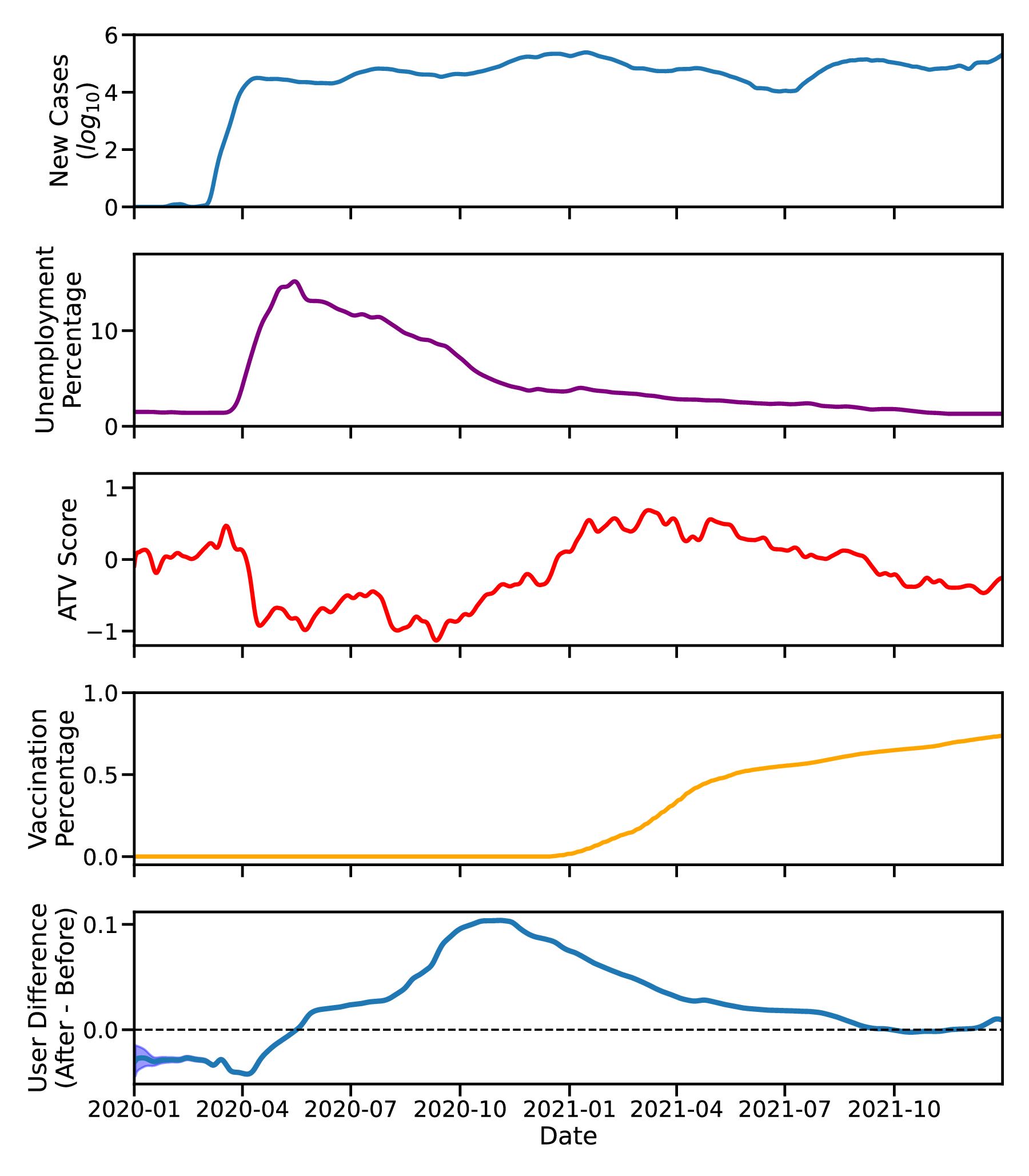

Supplement: S5 Fig — (TIFF) [file pone.0308122.s005.tiff]

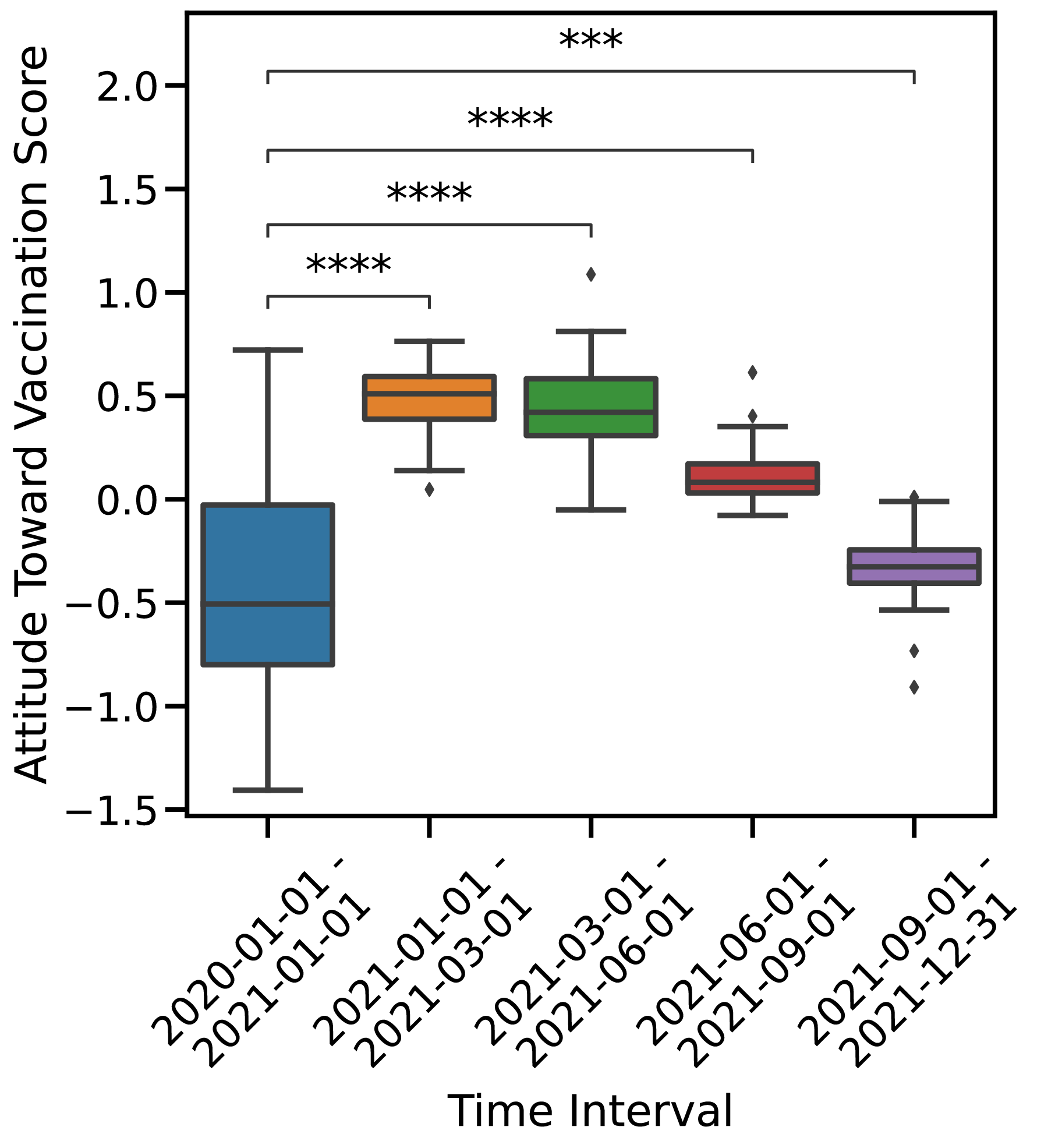

Supplement: S6 Fig — To accommodate the lack of tweets in 2020, we plotted the ATV scores for that year in a single box plot until 2021. The Mann-Whitney U test was used to calculate the statistical significance of each box plot with Bonferroni correction. (TIFF) [file pone.0308122.s006.tiff]

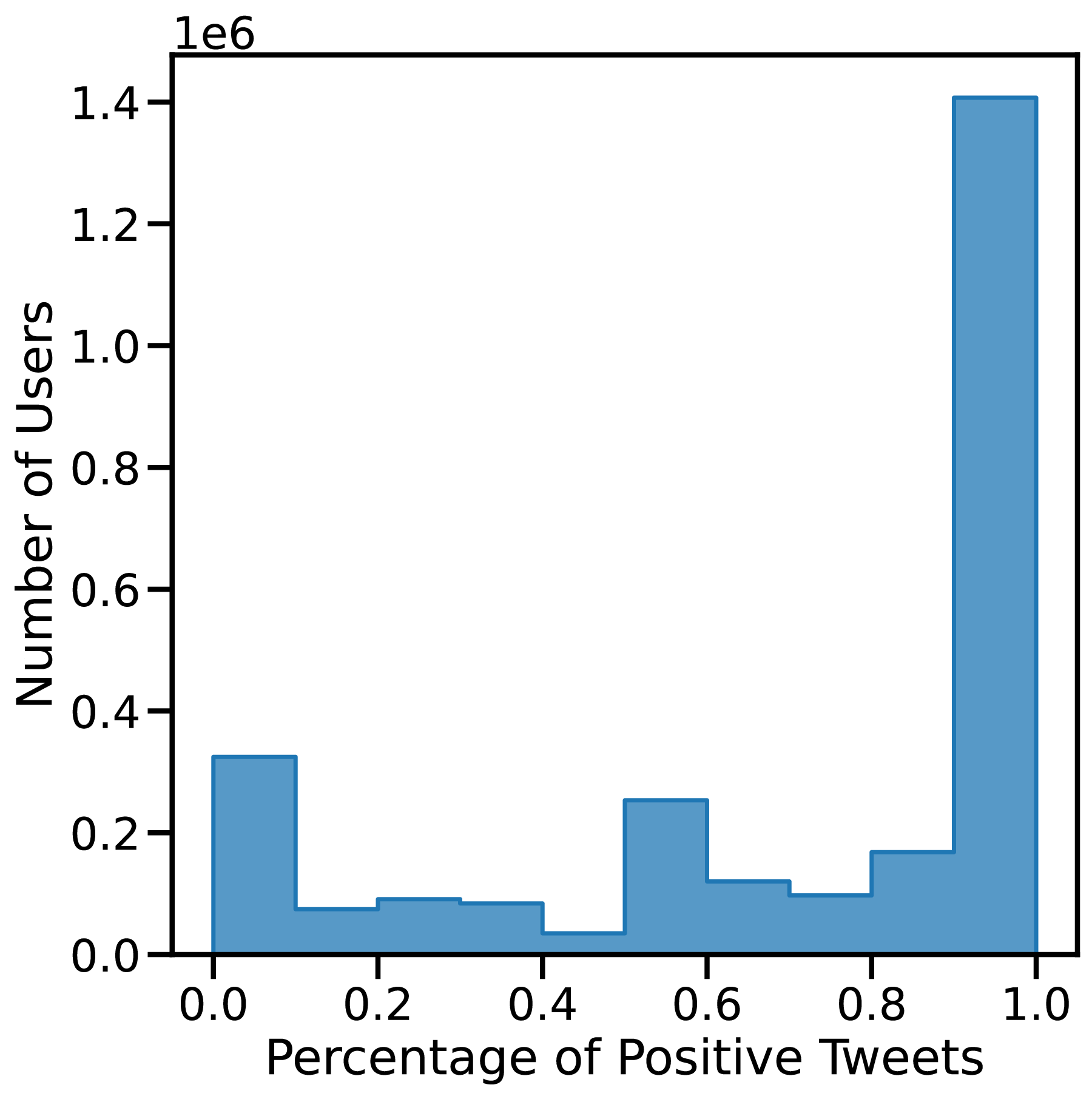

Supplement: S7 Fig — (TIFF) [file pone.0308122.s007.tiff]

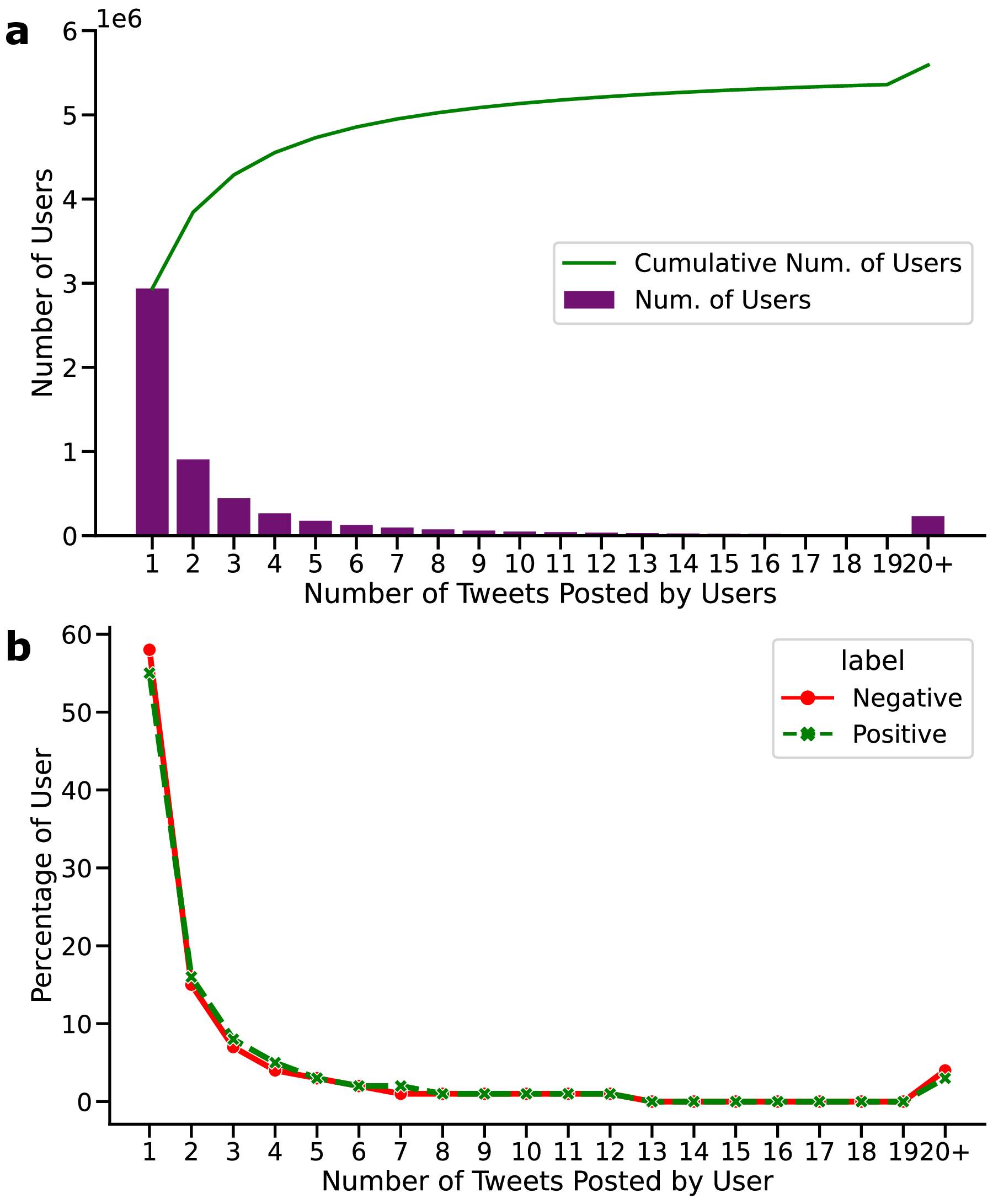

Supplement: S8 Fig — Approximately, half of the users sent only one tweet, while the other half sent multiple tweets. B: The percentage of users based on the number of tweets they posted by tweet attitude. It reveals that users with negative and positive attitudes did not differ in terms of the number of tweets they posted. (TIFF) [file pone.0308122.s008.tiff]

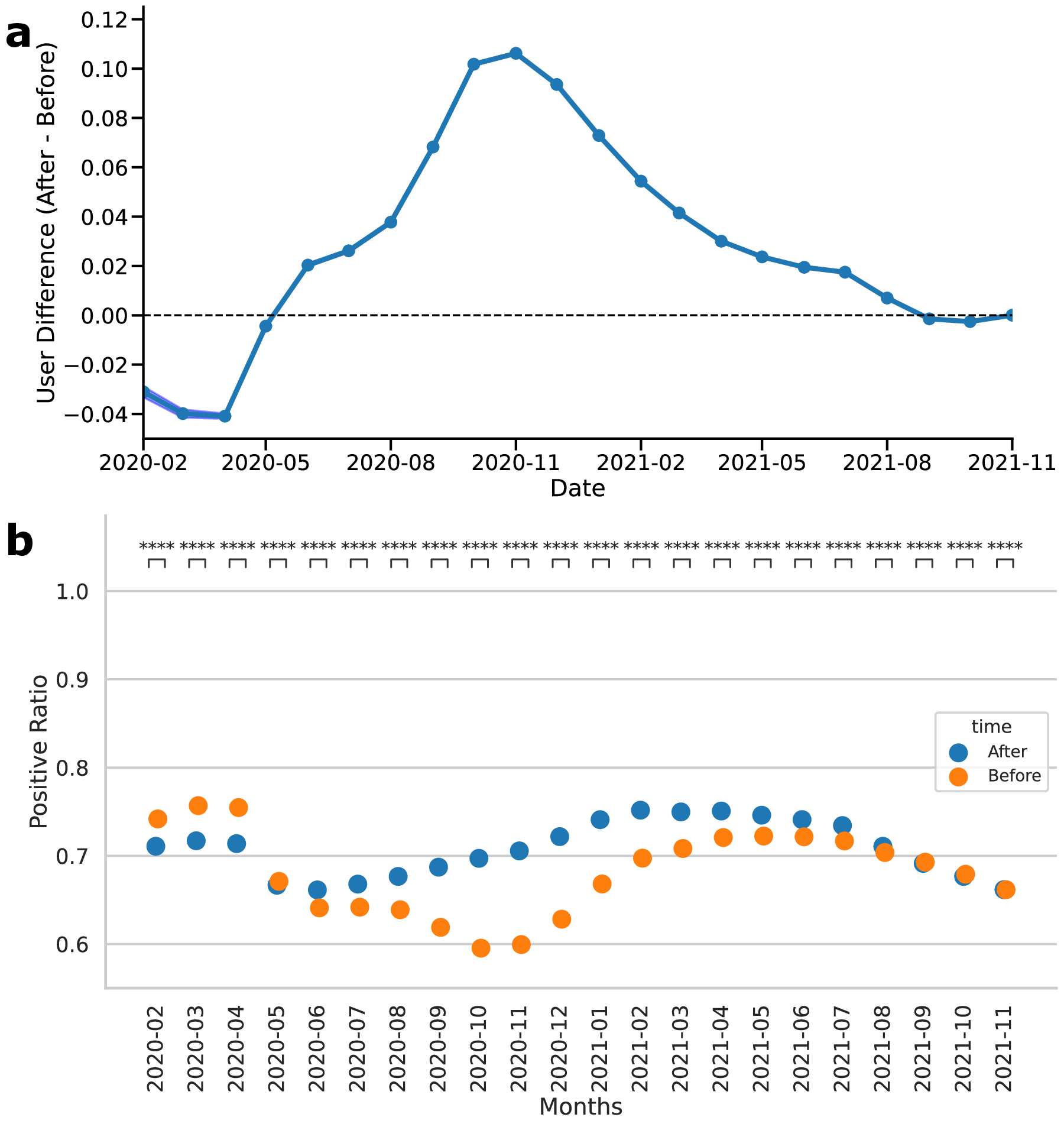

Supplement: S9 Fig — The figure includes two parts: A: percentage of users’ attitude change in each month, with points on the line indicating the change of average attitude towards a more positive (higher values) or negative attitude (lower values) after each month; and B: the average monthly positive ratio of each user, with changes before and after each month presented. In November 2020, there was a roughly 11% shift towards a positive attitude. We calculated the p-values using the Wilcoxon Test and corrected them using the Bonferroni method. (TIFF) [file pone.0308122.s009.tiff]

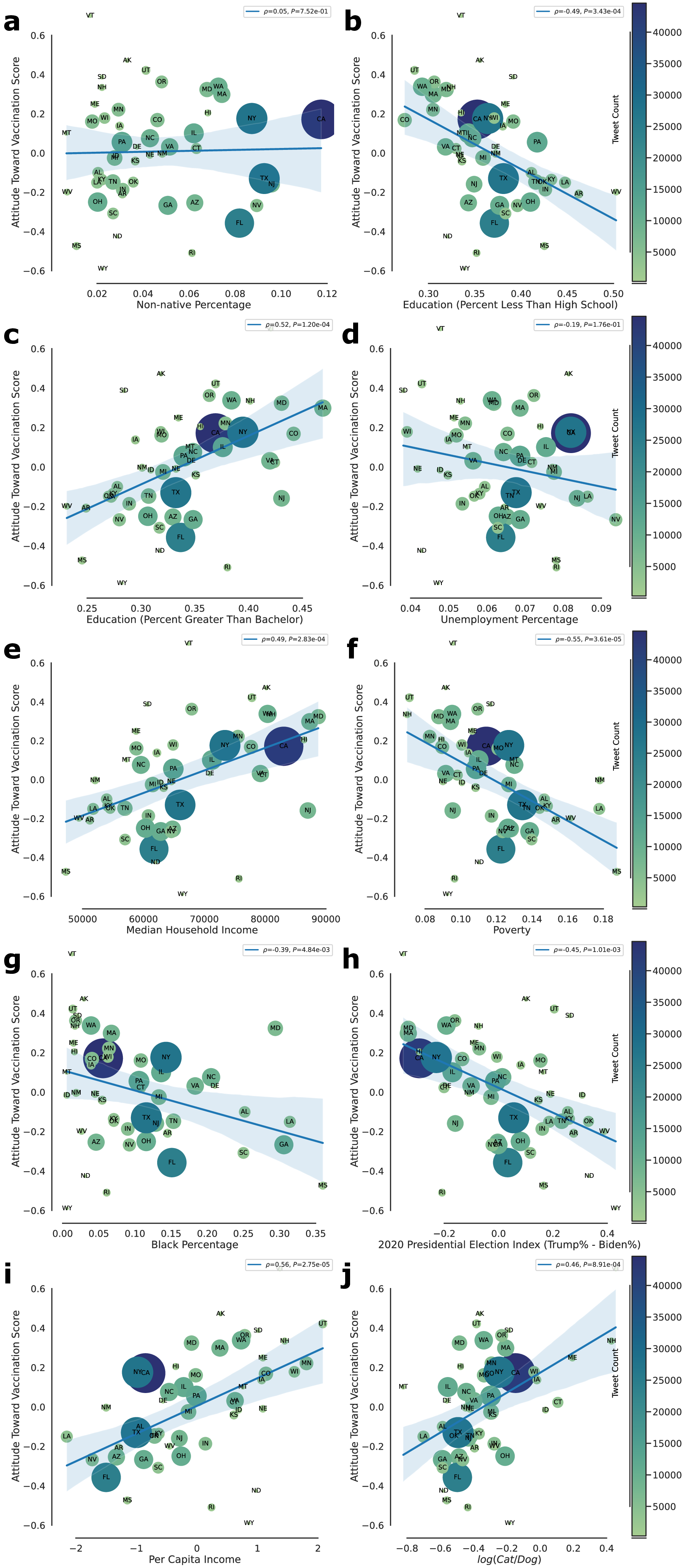

Supplement: S10 Fig — (TIFF) [file pone.0308122.s010.tiff]

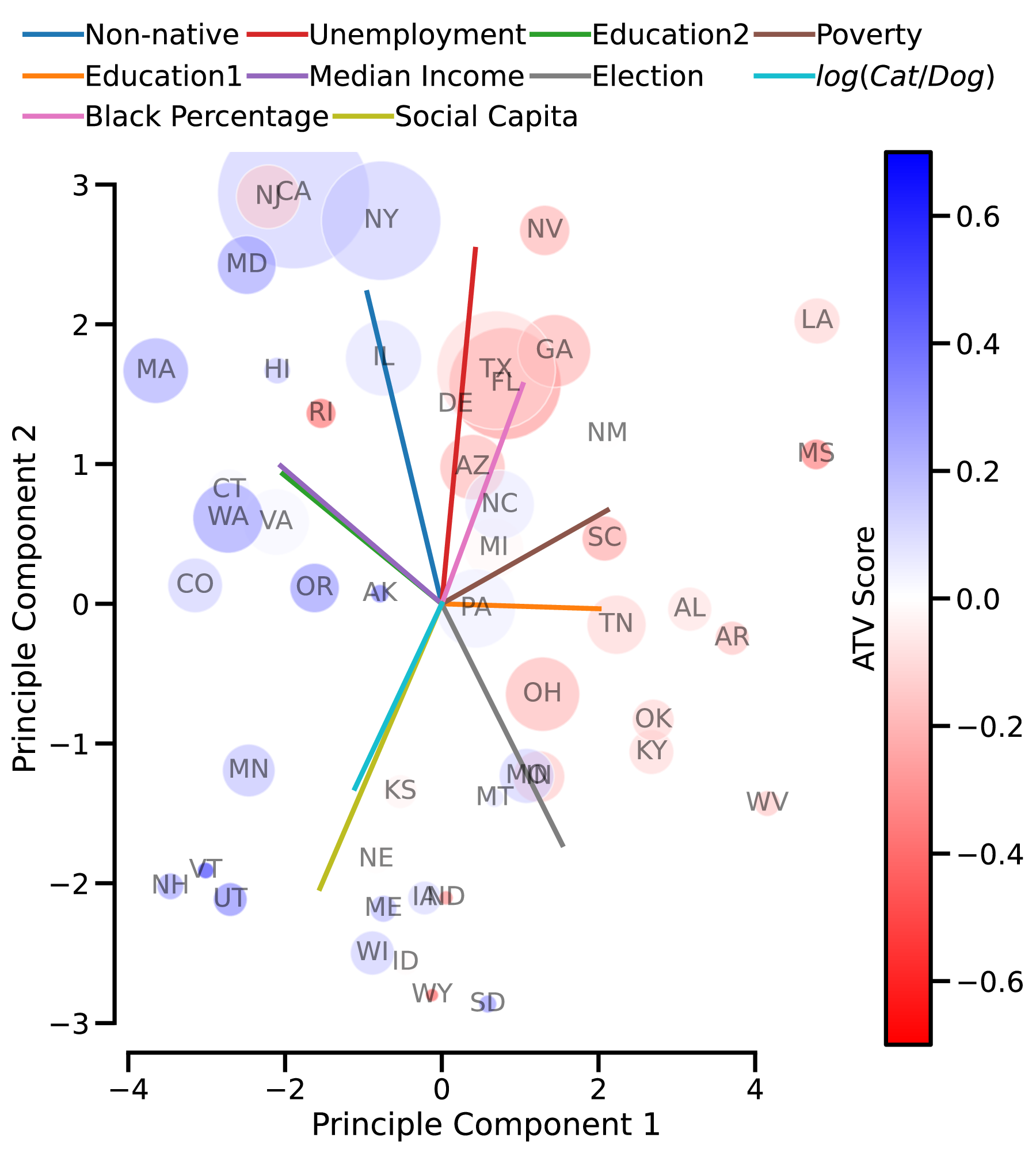

Supplement: S11 Fig — (TIFF) [file pone.0308122.s011.tiff]

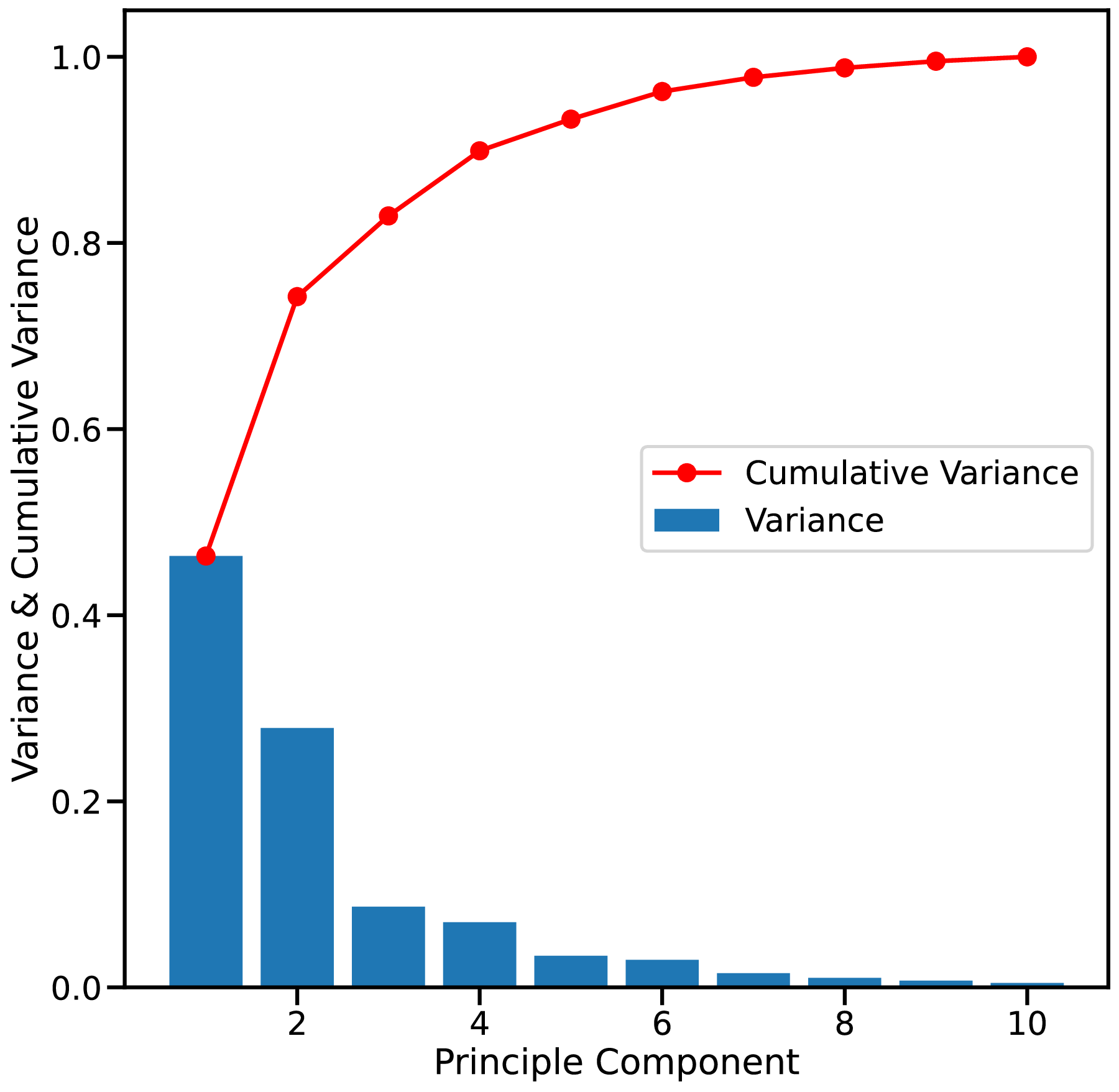

Supplement: S12 Fig — Over 75% of the variance is explained by the top two principal components. (TIFF) [file pone.0308122.s012.tiff]

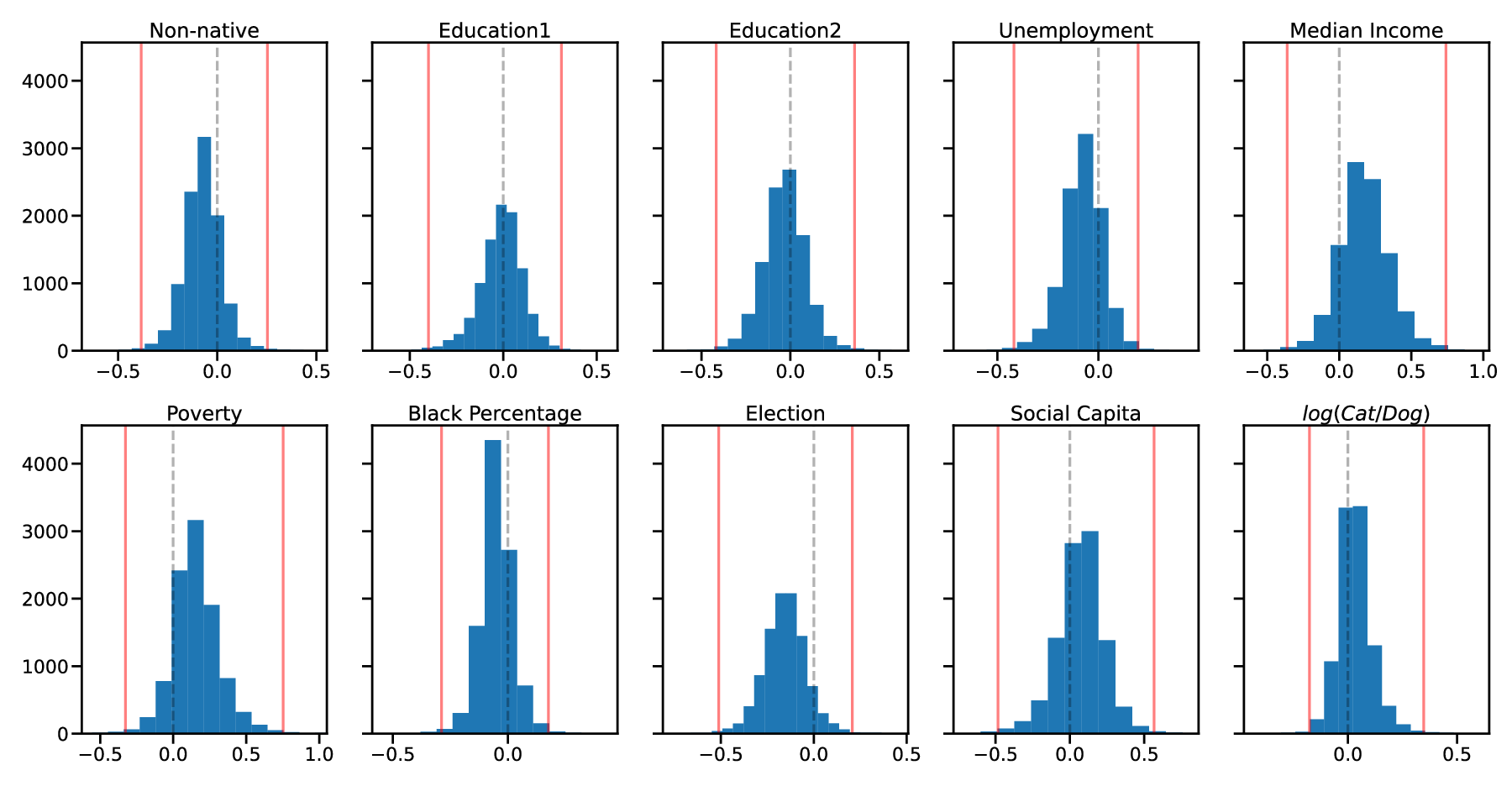

Supplement: S13 Fig — The figure includes a dashed line indicating the null hypothesis that the coefficient is zero, and red lines representing the confidence intervals for the coefficient. The null hypothesis (zero coefficient) is contained within these intervals. (TIFF) [file pone.0308122.s013.tiff]

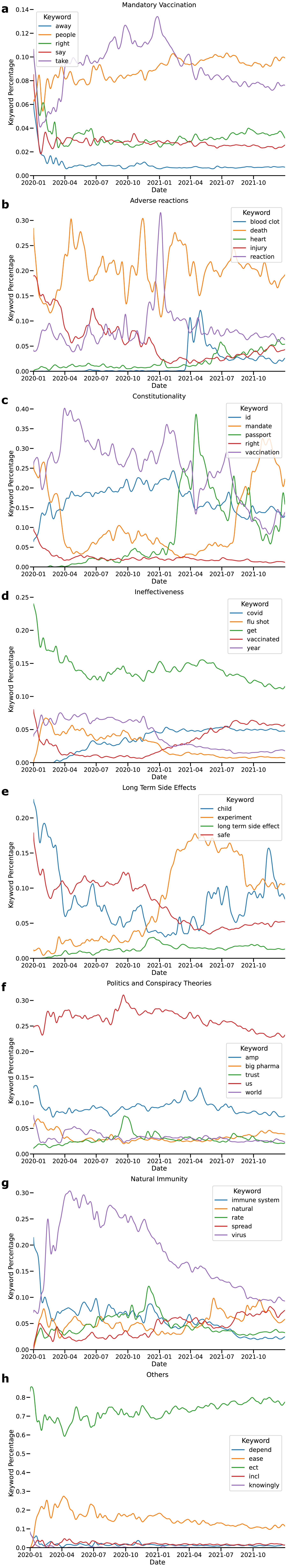

Supplement: S14 Fig — (TIFF) [file pone.0308122.s014.tiff]
